# Potassium Complexes of Quercetin-5′-Sulfonic Acid and Neutral O-Donor Ligands: Synthesis, Crystal Structure, Thermal Analysis, Spectroscopic Characterization and Physicochemical Properties

**DOI:** 10.3390/ma14226798

**Published:** 2021-11-11

**Authors:** Urszula Maciołek, Ewaryst Mendyk, Małgorzata Kosińska-Pezda, Daniel M. Kamiński, Anna E. Kozioł

**Affiliations:** 1 Institute of Chemical Sciences, Faculty of Chemistry, Maria Curie-Sklodowska University, 20-031 Lublin, Poland; ewaryst.mendyk@o2.pl (E.M.); daniel.kaminski@umcs.pl (D.M.K.); anna.koziol@poczta.umcs.lublin.pl (A.E.K.); 2 Faculty of Chemistry, Rzeszow University of Technology, 35-959 Rzeszow, Poland; m.kosinska@prz.edu.pl

**Keywords:** quercetin-5′-sulfonic acid, polymeric potassium complexes, acetone solvate, DMSO solvate, crystal structure, thermal analysis, solubility, spectroscopic studies

## Abstract

The coordination ability of QSA^−^ ligand towards potassium cations was investigated. Potassium complex of quercetin-5’-sulfonate of the general formula [KQSA(H_2_O)_2_]_n_ was obtained. The [KQSA(H_2_O)_2_] (**1**) was a starting compound for solvothermal syntheses of acetone (**2**) and dimethylsulfoxide (**3**) complexes. For the crystalline complexes **1**–**3**, crystals morphology was analyzed, IR and Raman spectra were registered, as well as thermal analysis for 1 was performed. Moreover, for **1** and **3,** molecular structures were established. The potassium cations are coordinated by eight oxygen atoms (KO_8_) of a different chemical nature; coordinating groups are sulfonic, hydroxyl, and carbonyl of the QSA^−^ anion, and neutral molecules—water (**1**) or DMSO (**3**). The detailed thermal studies of **1** confirmed that water molecules were strongly bonded in the complex structure. Moreover, it was stated that decomposition processes depended on the atmosphere used above 260 °C. The TG–FTIR–MS technique allowed the identification of gaseous products evolving during oxidative decomposition and pyrolysis of the analyzed compound: water vapor, carbon dioxide, sulfur dioxide, carbonyl sulfide, and carbon monoxide. The solubility studies showed that **1** is less soluble in ethanol than quercetin dihydrate in ethanol, acetone, and DMSO. The exception was aqueous solution, in which the complex exhibited significantly enhanced solubility compared to quercetin. Moreover, the great solubility of **1** in DMSO explained the ease of ligand exchange (water for DMSO) in [KQSA(H_2_O)_2_].

## 1. Introduction

Flavonoids are natural compounds originating from various species of plants. They possess different bioactive functions: anti-microbial, antioxidant, antitumor, neuroprotective, and cardioprotective. It is worth emphasizing that among other beneficial health effects of flavonoids, their greatest impact has been observed in the anticancer field [1,2,3,4].

**Scheme 1:**
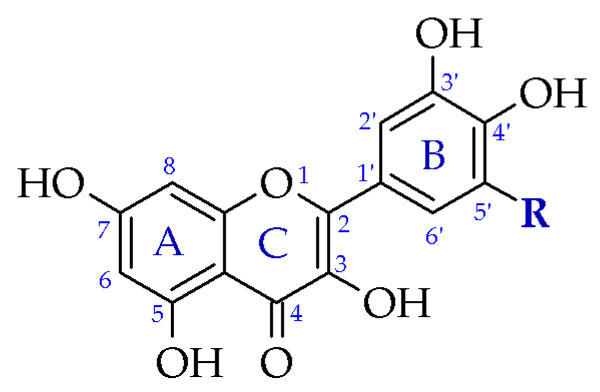
Structure of quercetin (R = H) and quercetin-5′-sulfonic acid (R = SO_3_H).

Quercetin (QUE; 3,3′,4′,5,7-pentahydroxyflavone, Scheme 1) is one of the best known and practically used flavonoids, therefore it is also the most thoroughly examined. The compound occurs naturally in many fruits and vegetables, for example in lettuce, peppers, onions, broccoli, capers, grapes, strawberries, cherries, mangoes, and blueberries. It has been proven that quercetin has antioxidant, anti-inflammatory, antimicrobial, antineoplastic, neuroprotective, and antiallergic properties [5,6,7,8,9]. For this reason, quercetin has gained widespread approval and is extensively used in the healthcare industry, not only as a dietary supplement but also as an adjuvant medicine [10].

A literature review shows that the chemical modification of flavonoids, e.g., through their metal complexation, is a new promising new trend for drug delivery systems characterized by better pharmacological activities and clinical profiles than the parent flavonoids [1]. Nevertheless, the challenge and limitation in pharmacological use of the unmodified flavonoids is their poor bioavailability due to the incredibly low water solubility [1]. This is why the studies concerning the increase in flavonoids solubility through chemical modification, co-crystallization, as well as metal complexation, is undoubtedly one of the most promising ways to achieve the goal. One of the methods to improve the solubility of the flavonoids in water is the synthesis of sulfonic derivatives of flavonoids [11,12,13,14,15]. This significantly extends their application, without increasing the toxicity of the compound.

So far, several papers have reported the crystal structures of sulfonic derivatives of different flavonoids and their complexes with alkali and transition metals (Table 1) [16,17,18,19,20,21,22,23,24,25,26,27,28,29,30,31,32,33,34]. The pattern of cation–anion interactions in these complexes depends on the position of the substituted sulfonic group and on the metal cation. The sulfonic group is not always a part of the metal coordination sphere. Moreover, some cations prefer to coordinate water molecules and form mononuclear complexes. It should be noted that ligands in the known complexes are derivatives containing sulfonic groups in the positions 6 [21,22,23,24,25,26,28], 8 [17,18,19,20] and 5′ [27] of the flavonoid skeleton (Table 1). Until now, the structure of only one complex with potassium cation has been determined [19]. It should be assumed that the structure of the complex in the solid phase, i.e., the type of ligands, the mode of metal cation coordination, the structure of coordination centers (single-core, multi-core, polymeric complex), affect the solubility of the compound. Among the compounds listed in Table 1, none has such properties tested.

As for other unmodified flavonoids, the applications of quercetin in its parent form are limited by the poor bioavailability caused by low solubility in water [10,16]. To increase the solubility of quercetin, the encapsulation as well as chemical modification were used [10]. Considering the known chemical modification methods, sulfonation of quercetin (Scheme 1) is frequently used. Sodium quercetin-5′-sulfonate is one of the better-tested derivatives of quercetin [11,12,13,14,15,17,35]. However, the product of the reaction of quercetin-5′-sulfonic acid with potassium (K^+^) ions has been poorly investigated.

In a previous paper, initial physicochemical characteristics of the potassium quercetin-5′-sulfonate (KQSA) have been presented and its anticancer and the antioxidant properties have been tested [36]. Recently, using the solvothermal synthesis, crystals of KQSA complexes with additional neutral *O*-donor ligands (acetone and DMSO) were obtained. Herein, we present the investigation and detailed analysis of the crystal structures of new K^+^ complexes with quercetin-5′-sulfonic acid. The presented studies show that the compound has an organic ligand-cation complex structure, rather than a simple ionic salt as reported previously [11,12,13,14,15,35]. Thermal analysis is commonly used in the investigations on biologically active compounds to estimate purity, thermal stability, shelf life, as well as mechanisms of decomposition. It is also important to check the possibility of utilizing the compound’s waste with a method that is not harmful to the environment, e.g., whether incineration is the correct method [37,38,39,40]. Thus, advanced thermal analyses of KQSA were also performed. Moreover, the solubility in selected solvents and spectral properties of KQSA were reported in this paper.

## 2. Materials and Methods

### 2.1. Materials

Quercetin dihydrate (C_15_H_10_O_7_∙2H_2_O) was purchased from Lachema & Oxford Vitality. Potassium hydroxide, sulfuric acid, ethanol, and acetone were acquired from POCH, Poland, while HPLC grade methanol and DMSO were purchased from Honeywell. All the chemicals and solvents were of analytical grade and used without further purification.

### 2.2. Synthesis of KQSA Complexes

The quercetin-5′-sulfonic acid (HQSA) and its complex with potassium (KQSA) were synthesized as described previously [36,41]. Single crystals of [KQSA(H_2_O)_2_] (**1**) were obtained via recrystallization of the yellow microcrystalline powder from water–ethanol (1:1 *v/v*) solution.

Subsequently, 20 mg of compound **1** and 3 mL of solvent (acetone, **2** or DMSO, **3**) were placed into 5 mL screw-cap Teflon vials and tightly sealed. Solvates of complex **2** and **3** were obtained at 72 °C by heating the vial on the hot stage in two modes: for one week (**2**) and for three weeks (**3**). In these conditions, water molecules from the K^+^ coordination sphere of **1** were replaced by the molecules of the used solvent.

### 2.3. Methods

The single-crystal X-ray diffraction data were collected on a Rigaku diffractometer (Rigaku, Tokyo, Japan) with CuKα radiation (λ = 1.54184 Å) for **1** at 293 K, while those for **3** at 150 K. Cell refinement and data collection as well as data reduction and analysis were performed with the CrysAlisPro 1.171.39.27b (Rigaku Oxford Diffraction, Tokyo, Japan) [42]. Structures were solved applying direct methods with the use of the SHELXS-86 program and refined with the SHELXL−2018/3 [43,44,45]. All non-hydrogen atoms were refined with anisotropic displacement parameters. The hydrogen atoms were positioned either on the electron difference maps or were calculated from the geometry at idealized positions, depending on the quality of the crystal and diffraction data. The experimental details and final atomic parameters for **1** and **3** have been deposited with the Cambridge Crystallographic Data Centre (ID CCDC: 2114454, 2114455).

The morphology of the crystals **1**–**3** was examined using: (i) an optical microscope Nikon Eclipse MA200 (Nikon, Tokyo, Japan); images of crystals have been registered in reflected polarized light using the EDF deep focus technique; the Nikon NIST Elements BR software (version 3.21.03) was used to record and process the images. (ii) a Quanta 3D FEG scanning electron microscope (FEI Comp. Hillsboro, OR, USA). The sample was evenly distributed on SEM specimen stubs with a double adhesive carbon tape. Before imaging, samples were covered with a thin layer of Pd/Au using a Polaron SC7640 vacuum sputter coater (Quorum Technologies Ltd., East Sussex, UK). The micrographs were obtained in a high vacuum mode with an accelerating voltage of 5 kV and an Helios ETD (SE) detector (FEI Co. Hillsboro, OR, USA).

Infrared and Raman spectra were acquired with a Nicolet 8700 FT-IR/NXR 9650 FT-Raman spectrometer (Thermo Scientific, Waltham, MA, USA) using a Smart Orbit™ diamond ATR attachment, a 1064 nm Nd:YVO_4_ diode laser, and the LN_2_-cooled NXR Genie detector, respectively. For each spectrum, 64 (IR) and 1024 (Raman) scans were averaged. The Omnic software (version 8.1) was used to collect and process the spectra.

Simultaneous TG-DSC curves were performed on Netzsch STA 449 Jupiter F1 analyzer (Erich NETZSCH GmbH & Co. Holding KG, Selb, Germany) using corundum crucibles. The experiments were conducted in air and helium atmospheres with a 50 mL min^−1^ flow rate and a heating rate of 10 °C min^−1^ in the temperature range of 30–1450 °C. An empty alumina crucible was used as a reference. The mass of the samples used for this study was about 5 mg. The FTIR and MS spectra of gaseous products, which evolved during thermal decomposition of KQSA, were recorded in helium and air atmospheres at a heating rate of 10 °C min^−1^. The instruments used were a Netzsch STA 449 Jupiter F1 analyzer coupled with a Bruker Tensor FT-IR spectrometer (Bruker Corp., Billerica, MA, USA), and a Netzsch QMS 403D Aëolos mass spectrometer (Erich NETZSCH GmbH & Co. Holding KG, Selb, Germany). The FTIR spectra were acquired in the range 4000–600 cm^−1^ with a resolution of 4 cm^−1^. Mass spectra were recorded in the mass range of 10–300 amu. Data were collected and edited using the NETZSCH Proteus^®^ ver. 6.1.

A VT XRPD method was used to examine the crystalline phases. The diffraction patterns were collected on an Empyrean diffractometer with the PIXcel^3D^ detector (PANalytical, Almelo, The Netherlands) using monochromated Cu Kα radiation (λ = 1.54184 Å) in the 2θ range of 4.7–90° with a step of 0.026°. Heating temperature was controlled in an XRK 900 reactor chamber (Anton Paar, Graz, Austria). The samples were heated dynamically in air and helium atmospheres with a heating rate of 10 °C min^−1^ in the range of 25–800 °C. The ICDD PDF4 + 2021 diffraction database was used to identify the crystalline phases.

The solubility of quercetin dihydrate and [KQSA(H_2_O)_2_] in distilled water, ethanol, acetone, and DMSO at 20 °C was studied by saturation solubility technique [46]. For this purpose, the saturated solutions of the tested compounds were prepared in volumetric flasks and each of them was treated with 10 cm^3^ of the appropriate solvent. The contents of the flasks were stirred on a magnetic stirrer for 12 h at 20 °C, then left for another 12 h, and filtered eventually. The standard stock solutions in each solvent were prepared and diluted to obtain a concentration range of 1–25 μg/mL (for QUE·2H_2_O, and **1** in ethanol, acetone and DMSO), 0.1–4 μg/mL (for **1** in water) or 0.1–1.5 μg/mL (for QUE·2H_2_O in water). The absorbance of the resulting solutions was measured using a UV-VIS-NIR V-670 (Jasco, Tokyo, Japan) spectrophotometer against the corresponding blank to obtain calibration curves. The compound’s solutions follow Beer–Lambert’ Law over the mentioned concentration range at the selected wavelength with R^2^~1. The maximum solubility of QUE·2H_2_O and **1** in ethanol, acetone, DMSO, and water was determined using the above standard calibration curves. The concentration of the compounds in bulk saturated solutions was determined by substituting the absorbance and absorptivity values in the Beer–Lambert Law equation.

## 3. Results and Discussion

### 3.1. Morphology of Crystals

**Figure 1:**
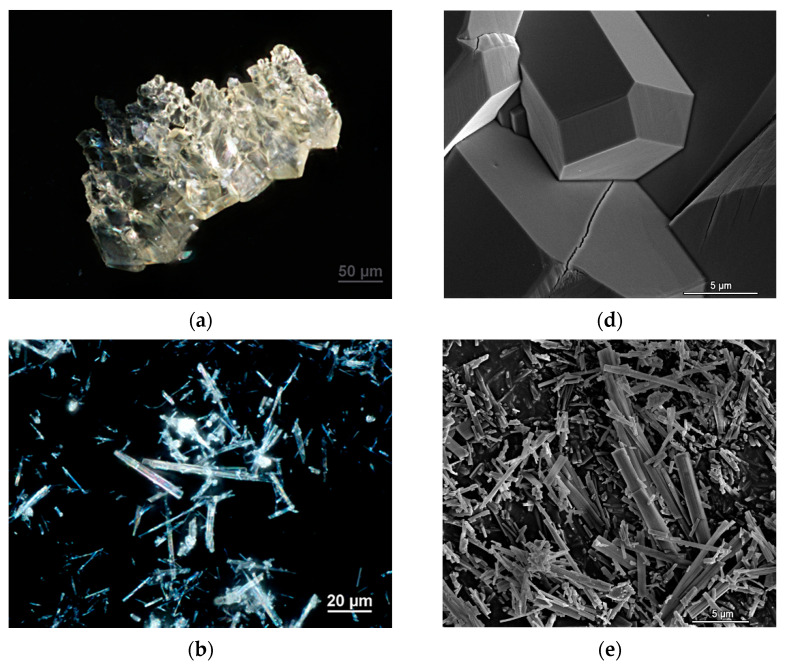

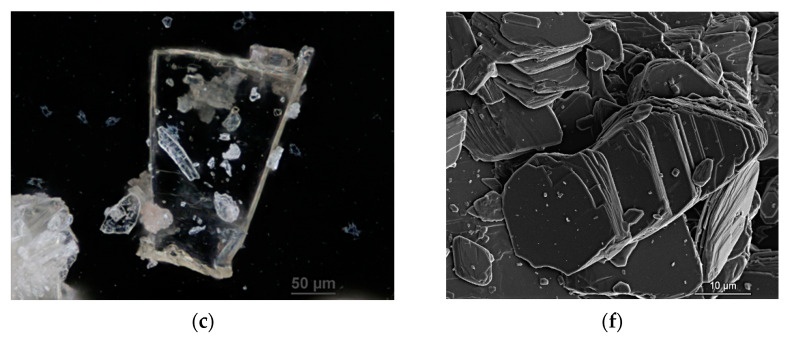
Images from an optical microscope (**a**–**c**) and SEM micrographs (**d**–**f**) at low voltage (5 kV) for crystalline complexes **1** (**a**,**d**), **2** (**b**,**e**), and **3** (**c**,**f**), respectively.

Using solvothermal synthesis, in complex **1** [KQSA(H_2_O)_2_]_n_, neutral ligand–water molecules were exchanged with acetone (**2**) and DMSO (**3**) molecules, respectively. Complexes **1** and **3** formed single crystals suitable for X-ray diffraction studies, while complex **2** formed only a very unstable microcrystalline phase. Images from an optical microscope and SEM micrographs of crystals **1**–**3** are presented in Figure 1. Compound **1** crystallizes in the form of prisms (Figure 1a,d), **2** forms very fine needles with large part of debris crystals (Figure 1b,e) that undergo rapid desolvation, while solid complex **3** consists of large aggregates of lamellar crystals (Figure 1c,f).

### 3.2. Crystal Structure of Compound ***1***

Molecular and crystal structure of complex **1** (diaqua-potassium quercetin-5′-sulfonate) was determined using a single crystal X-ray analysis.

Crystal data for **1**: formula C_15_H_13_KO_12_S, Mw = 456.41, crystal system triclinic, space group P $\bar{1}$, unit cell dimensions a = 8.491(2) Å, b = 10.110(3) Å, c = 10.466(3) Å, α = 74.06(2)°, β = 87.57(2)°, γ = 82.99(2)°, V = 857.4(4) Å^3^, Z = 2, D(calc) = 1.768 g/cm^3^, μ = 4.519 mm^−1^, F(000) = 468. Crystal size 0.2 × 0.11 × 0.05 mm; range of θ = 21.436 to 68.390°, index ranges −10 ≤ h ≤10, −12≤ k ≤12, −12 ≤ l ≤12. The data were corrected for Lorentz and polarization effects. A multi-scan absorption correction was applied. Reflections collected/independent 10444/2897 [R(int) = 0.0231], parameters refined 298, reflections observed 2607. Goodness-of-fit on F^2^ = 1.065, final indices for [I > 2σ(I)] R1 = 0.0293, wR2 = 0.0796, and for all data R1 = 0.0328, wR2 = 0.0812. ∆p max/min 0.32 and −0.24 e Å^−3^. CCDC Number: 2114454.

**Figure 2:**
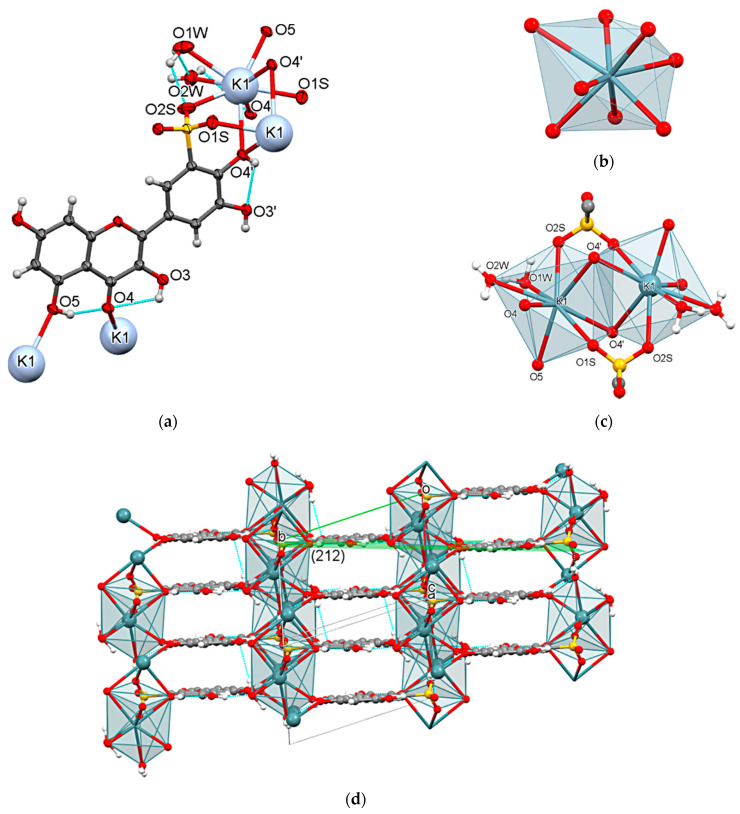
View of the crystal structure of **1**. (**a**) The K^+^ cation…QSA¯ anion interactions. Dashed lines indicate intramolecular O-H…O hydrogen bonds. (**b**) Coordination sphere of K^+^ cation. (**c**) The K_2_O_14_ dimer. (**d**) The planes of aromatic rings of the QSA¯ anion are parallel to the crystallographic plane (212).

In complex **1**, the QSA^−^ anion is a hexadentate O-donor ligand. The coordinating groups around K^+^ are sulfonic, hydroxyl, and carbonyl, wherein the O4’H hydroxyl and the sulfonic groups are chelating moieties (Figure 2a and Table 2). With two water molecules completing the sphere, the coordination number for K^+^ is equal to 8 (Figure 2b); further, around the center of symmetry, a dimer of spheres is formed with a common edge O4′...O4‘ (Figure 2c). Each of the cations forms the coordination K...O bonds with four anions (Table 2). This pattern of solid-state coordination results in the formation of a three-dimensional coordination polymer stabilized by both K...O bonds and O-H...O hydrogen bonds (Table 3). The anion hydroxyl groups and water molecules are hydrogen bond donors, whereas the oxygen atoms of anion and water are both the acceptors.

In the crystal structure of **1**, the coordination polyhedra K_2_O_14_ lie in the lattice plane (010), while the ligand aromatic rings are parallel to the plane (212) (Figure 2d). Such an orientation of the rings, with the distance of 3.398(2) Å, results in the structure stabilization by π…π stacking.

### 3.3. Thermal Analysis of Complex ***1***

**Figure 3:**
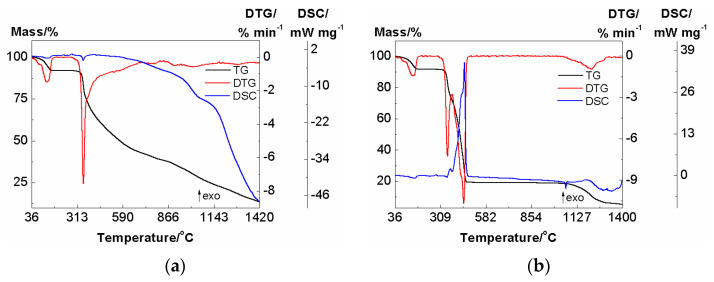
TG-DTG-DSC curves for complex **1:** (**a**) in helium (5.06 mg), (**b**) in air (5.00 mg).

In the present paper, thermal analysis was performed only for complex **1,** which is stable at room temperature. The simultaneous thermogravimetry–derivative thermogravimetry (TG-DTG) analysis and differential scanning calorimetry (DSC) were performed in flowing air and helium atmospheres to verify chemical composition of the [KQSA(H_2_O)_2_] and explore the thermal stability and decomposition paths. The TG–DTG-DSC curves registered in both atmospheres are presented in Figure 3a,b, respectively. The details of the thermal decomposition stages (temperature range, mass loss and peak temperature) are shown in Table 4.

**Figure 4:**
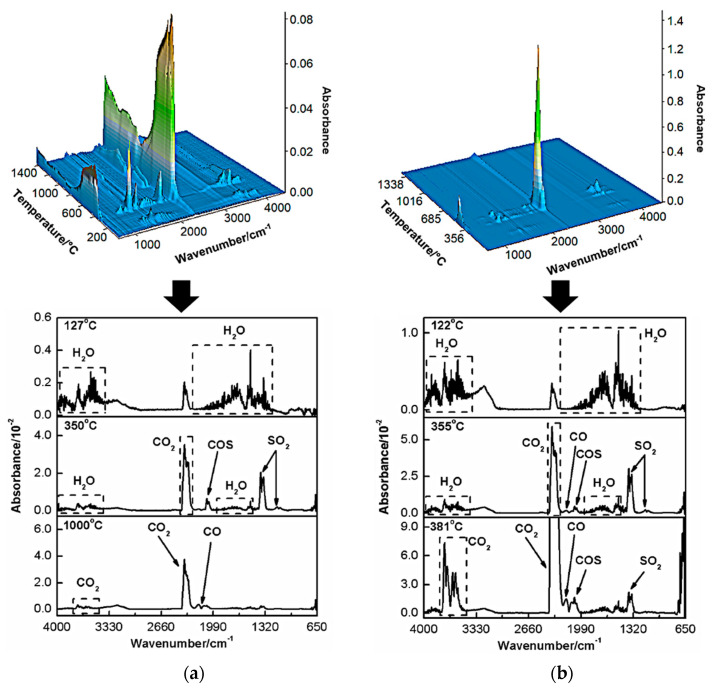
The 3D-2D FTIR spectra of gas products released during thermal decomposition of **1** in helium (**a**) and in air (**b**).

**Figure 5:**
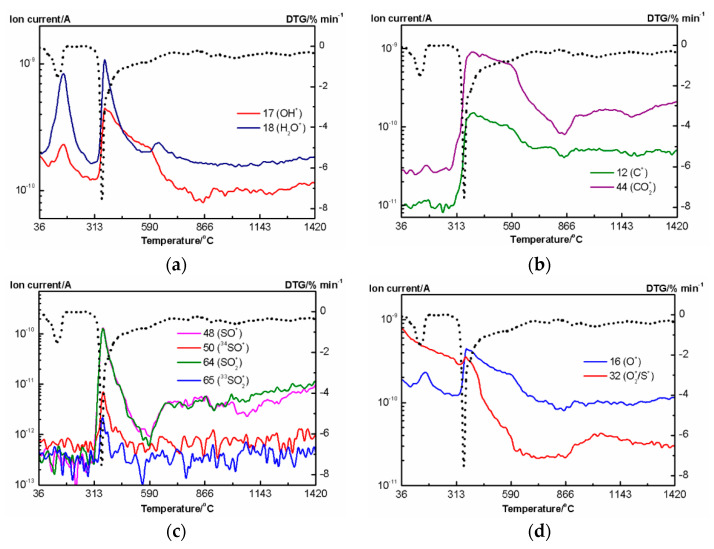
The ionic masses of gaseous products released during the thermal decomposition of **1** in helium and the corresponding ionic current tracks (the solid line) and a DTG run (the dotted line) related to the evolution of water (**a**), carbon oxides (**b**), sulfur dioxide (**c**) or selected species (**d**)**.**

**Figure 6:**
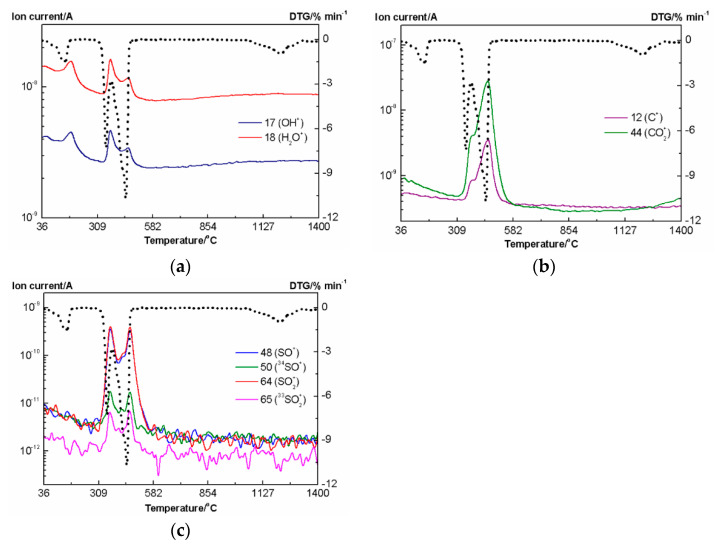
The ionic masses of gaseous products released during the thermal decomposition of **1** in air and the corresponding ionic current tracks (the solid line) and a DTG run (the dotted line) related to the evolution of water (**a**), carbon oxides (**b**) or sulfur dioxide (**c**).

The results of thermogravimetric analysis were additionally supported by the registration of temperature diffraction patterns (in the range 25–800 °C) in order to identify some solid crystalline intermediates. The combined TG-FTIR-MS techniques were also used to identify the evolved gaseous products of thermal decomposition of the complex. The FTIR spectra of the gases evolved at specific temperatures for the complex are shown in Figure 4. Ion currents for selected fragments with the corresponding DTG curve as a function of temperature are shown in Figure 5 and Figure 6.

Knowing that thermal degradation of the complex in air differs from that in helium, the results of each thermal analysis will be discussed separately.

#### 3.3.1. Thermal Analysis in Helium Atmosphere

Based on the TG–DTG-DSC curves registered in the helium atmosphere (Figure 3a), it can be concluded that the complex **1** is thermally stable up to about 50 °C. The first stage of its thermal degradation is observed in the range 50–200 °C on the DTG curve with a maximum rate at 127 °C and on the DSC curve as an endothermic peak with a maximum at 130 °C. The observed loss of mass is related to the dehydration process and its percentage (Δm_exp_ = 8.13%; Δm_calc._ = 7.89%) corresponds to the release of **2** moles of H_2_O. The TG-FTIR-MS analysis confirms the first stage of thermal decomposition of **1** to be dehydration, because of the signals with m/z 17 and 18 [47,48] in mass spectra (Figure 5a) as well as present bands corresponding to stretching and deformation vibrations of the OH groups of water molecules in the range 4000–3400 cm^−1^ and 1800–1200 cm^−1^ in FTIR spectra (Figure 4a) [49]. The temperature of dehydration above 120 °C indicates that water molecules are strongly bound in crystals [50]. Thus, it is consistent with the results of a single crystal X-ray analysis of **1**, which showed two water molecules coordinated to potassium ions.

**Figure 7:**
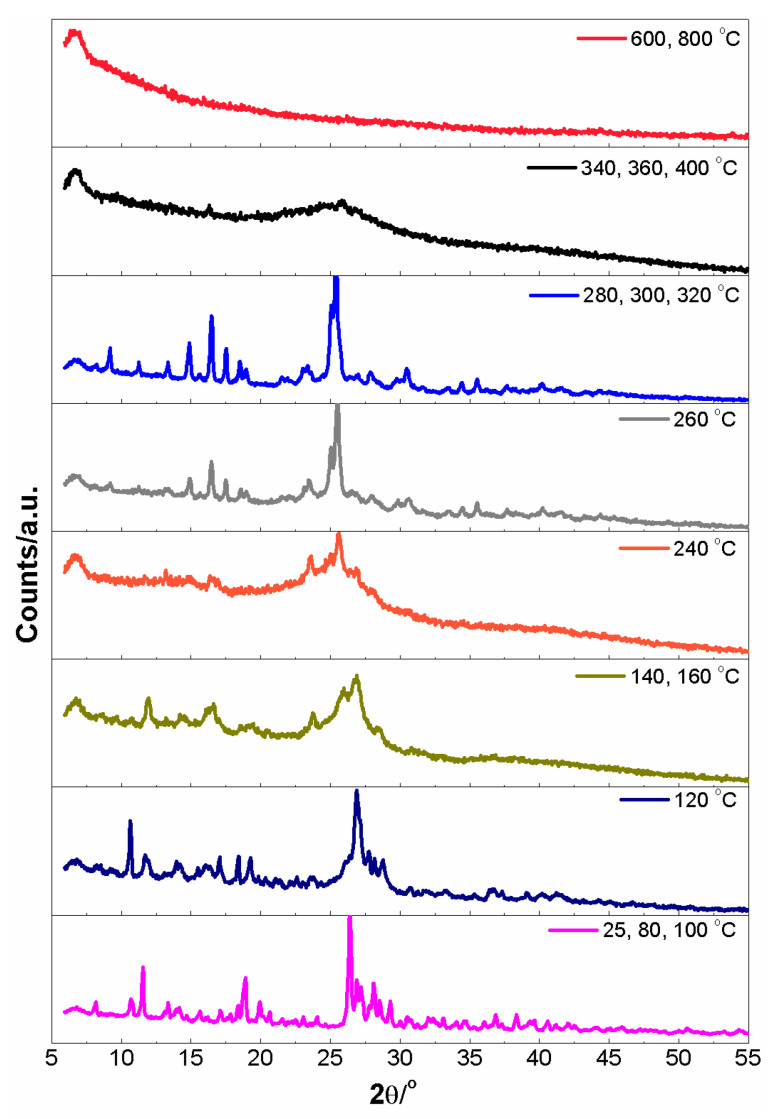
The powder XRD patterns of **1** after heating in the range of 25–800 °C in helium.

The temperature powder diffractograms (Figure 7) revealed that the loss of water results in the destruction of crystal **1**, since the high crystallinity of aqua complex decreases upon dehydration. Also, broadening of the diffraction peaks of a lower intensity in the XRD patterns are observed.

For the intermediate anhydrous compound, there is a plateau on the DTG curve up to about 300 °C, while the DSC curve shows a small exothermic peak at 280 °C corresponding to the crystallization of the new phase, which was confirmed by the temperature diffraction patterns (Figure 7).

Further heating to 300−700 °C causes extended degradation of an organic moiety of the anhydrous **1** with a distinguishable endothermic peak on the DSC at 350 °C and the formation of an inorganic amorphous intermediate (Figure 3a). The decomposition of the organic part of the complex corresponds to the release of H_2_O, CO_2_, SO_2_, and COS species. The presence of carbon dioxide is evidenced by bands from asymmetric stretching and bending vibrations in the ranges of 2400–2250 cm^−1^ and 750–600 cm^−1^ in FTIR spectra (Figure 4a), as well as signals with m/z 12 (C^+^), 16 (O^+^) and 44 (CO_2_^+^) in the mass spectrum (Figure 5) [35,48,49,51,52,53]. The release of SO_2_ confirms the presence of signals with m/z 48, 50, 64 and 65 in the mass spectra (Figure 5) and the presence of bands at 1400–1300 and 1250–1050 cm^−1^ in FTIR spectra attributed to asymmetric and symmetric stretching mode of S=O group, respectively (Figure 4a) [54]. The carbonyl sulfide was identified on the basis of characteristic bands at 2070 and 2050 cm^−1^ in the corresponding FTIR spectra (Figure 4a) [35,53,54].

Despite the failure of the experimental verification of the solid product obtained at 700 °C, the calculations (Table 4) and similarity of the results to those obtained previously for sodium complex [41] allow to assume that a mixture of potassium carbide and carbon black are intermediate products at 700 °C. The TG–DTG-DSC run (Figure 3a) shows that above 700 °C, the mass is still decreasing above 700 °C. It can be due to the slow thermal dissociation of K_2_C_2_ (K_2_C_2_ = 2K_(gas)_ + 2C) in an inert atmosphere. In the gaseous products released above 700 °C, only carbon oxides are present (Figure 4a and Figure 5). Contrary to the results obtained for sodium quercetin-5′-sulfonate [41], the pyrolysis of the mixture of potassium carbide and carbon black is not completed up to 1420 °C.

#### 3.3.2. Thermal Analysis in Air Atmosphere

Thermal studies of **1** performed in air in the temperature range of 25–300 °C show no significant differences in a thermal behavior compared to the results in the helium atmosphere. The first weight loss, which is observed on the DTG curve with a maximum at 144 °C and an endothermic peak in the DSC run with a maximum at 154 °C, is from the dehydration process. This was also confirmed by EGA analysis (Figure 4b and Figure 6). For the intermediate product, anhydrous potassium quercetin-5′-sulfonate, no changes related to weight loss to about 300 °C were observed. Moreover, the DSC shows a very small exothermic peak at 280 °C which, similar to the NaQSA complex [41], may be related to a sample recrystallization.

The influence of atmosphere on the mechanism of the thermal decomposition of the complex is visible above 300 °C. According to the DTG run in air (Figure 3b), the oxidative degradation of the organic part of anhydrous **1** takes place in two stages: at 351 and 449 °C, which correspond to two (weak and very strong) exothermic effects on the DSC curve, respectively. The EGA analysis revealed that the first oxidative step corresponds to the complete removal of the phenolic groups from the cationic coordination sphere, detachment of the sulfonic group, and a breakdown of the aromatic ring B of the QSA^−^ anion, together with the releases of small gas molecules: H_2_O, CO, CO_2,_ SO, SO_2_ and COS (Figure 4b and Figure 6). In the second stage, apart from the ring B disintegration, the breakdown of the aromatic rings A and C in the QSA¯ moiety also takes place.

**Figure 8:**
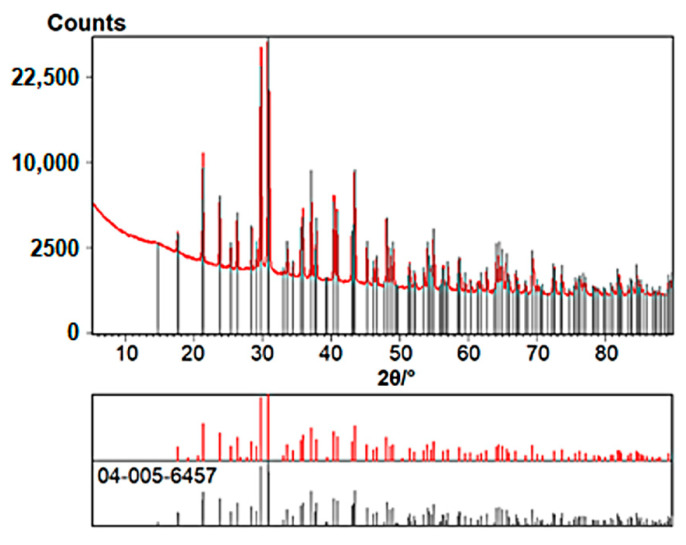
An identification of the decomposition product of **1** isolated from an experiment in air at 600 °C. The presence of potassium sulfate(VI) (Ref. Code 04-005-6457—K_2_SO_4_) was proved using the ICDD PDF4+2021 diffraction database.

The solid product obtained after the organic moiety degradation in air differs from that formed in helium atmosphere. Solid residue percentage at 600 °C (Table 4) as well as experimental data from a powder X-ray analysis (Figure 8) indicate the formation of crystalline potassium sulfate.

Continued heating results in the appearance of a sharp endothermic peak on the DSC curve with a maximum at 1062 °C (Figure 3b), resulting from the melting potassium sulfate. It is followed by a weight loss corresponding to a peak at 1215 °C on the DTG curve. The similarity of transformations during heating of solid potassium sulfate to those described for sodium sulfate [41] allows to assume that above 1000 °C, evaporation (K_2_SO_4(liquid)_ = K_2_SO_4(gas)_) and decomposition (K_2_SO_4(liquid)_ = 2K_(gas)_ + SO_2(gas)_ + O_2(gas)_) of the molten K_2_SO_4_ takes place.

### 3.4. Dissolution Tests of Complex ***1***

The solubility of quercetin dihydrate and complex **1** in water, acetone, ethanol, and DMSO of quercetin dihydrate and complex **1** was determined spectrophotometrically (Table 5).

The results of solubility studies clearly indicate that, compared to quercetin, complex **1** shows lower solubility in acetone, ethanol, and DMSO. Nevertheless, it was evidenced that the solubility of **1** in water is 45-fold increased with regard to quercetin. It should be noted that **1** dissolves in acetone and DMSO better than in water. This may be due to the increased hydrophobicity of acetone and DMSO compared to the water molecule, and the influence of the polarity of the solvent cannot be excluded. Thus, an intercalation of molecules of those solvents into solid networks can be facilitated.

### 3.5. Crystal Structure of Compound ***3***

Molecular and crystal structure of the complex **3** (monodimethyl sulfoxide bis(dimethyl sulfoxide) potassium quercetin-5′-sulfonate) was determined using a single crystal X-ray analysis.

Crystal data for **3**: formula C_42_H_54_K_2_O_26_S_8_, Mw = 1309.53, crystal system triclinic, space group P $\bar{1}$, unit cell dimensions a = 10.6363(7) Å, b = 11.9470(9) Å, c = 23.5105(9) Å, α = 91.402(8)°, β = 95.632(8)°, γ = 109.998(8)°, V = 2788.5(3) Å^3^, Z = 2 (Z′ = 2), D(calc) = 1.560 g/cm^3^, μ = 5.040 mm^−1^, F(000) = 1360. Crystal size 0.13 × 0.11 × 0.02 mm; range of θ = 3.786 to 71.256°, index ranges −12 ≤ h ≤ 12, −14 ≤ k ≤ 14, −28 ≤ l ≤ 28. The data were corrected for Lorentz and polarization effects. A multi-scan absorption correction was applied. Reflections collected/independent 43097/10138 [R(int) = 0.1271], parameters refined 728, reflections observed 6188. Goodness-of-fit on F^2^ = 1.029, final R indices [I > 2σ(I)] R1 = 0.0910, wR2 = 0.2337, R indices (all data) R1 = 0.1374, wR2 = 0.2627. ∆pΔρ max/min 1.07 and −0.72 e Å^−3^. CCDC Number: 2114455.

**Figure 9:**
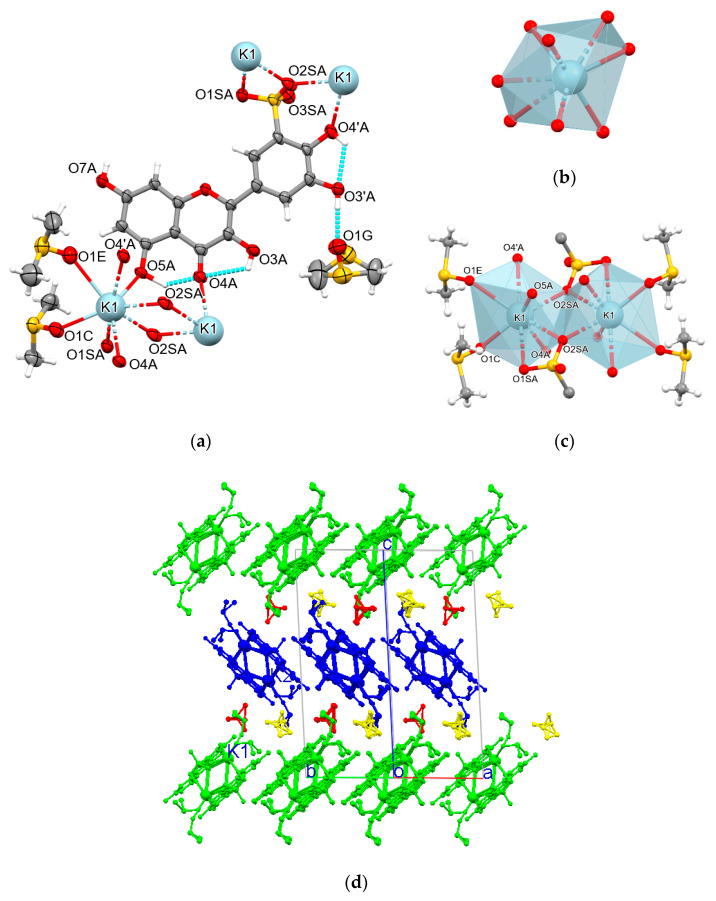
The crystal structure of 3. The structure of the polymeric complexes formed by the cations K(1) and K(2) is analogous, and in this figure only structure involving K(1) is shown. (**a**) The environment of the cation and QSA¯ anion with DMSO molecules coordinating K^+^ and solvating the crystal network. (**b**) Coordination sphere of K^+^ cation (LK = 8). (**c**) The centrosymmetric K_2_O_14_ dimer. (**d**) View of the crystal 3 down the direction [110]. The solvating disordered DMSO molecules are accentuated in yellow and red. The coordination sphere of the K(1) cation is illustrated in green, and that of the K(2) cation in blue. Hydrogen atoms are omitted for clarity.

The complex **3** crystallizes with two chemical units [KQSA(DMSO)_2_DMSO] in the asymmetric part of the crystal unit cell. There are two DMSO molecules in the coordination sphere of both cations—K(1) and K(2). Moreover, coordinating DMSO molecules of a larger volume and hydrophobicity than the water molecules present in complex **1** cause two additional DMSO molecules to be incorporated in the network (Figure 9a). The QSA¯ anions are hexadentate O-donor ligands. As was observed for complex **1** (Table 2), in **3,** each of the cations forms coordination K-O bonds with four anions (Table 6). The coordinating groups around K^+^ are sulfonic, hydroxyl, and carbonyl, wherein the two O-atoms of the sulfonic group and O4’H hydroxyl form double-chelating moieties (Figure 9 and Table 6). Each of the two K^+^ cations finally reaches the coordination number of 8 (Figure 9b), and then by transforming around the center of symmetry, a dimer of distorted coordination polyhedra (K_2_O_14_) with a common edge (O_sulfate_...O_sulfate_) is generated (Figure 9c).

The formation of three K-O coordination bonds with a sulfonic group (two chelating and one bridging), and the inclusion of two intercalating DMSO molecules in the network generate two separate linear polymers (Figure 9d). The structure of one-dimensional polymers is stabilized by O-H...O hydrogen bonds (Table 7). Both polymers formed with the participation of the K(1) and K(2) cations are oriented parallel to the [110] direction, but the type and number of hydrogen bonds are different for them. The DMSO molecules that coordinate to the cations are oriented towards the outside of the polymer chain, which gives the surface a hydrophobic character. The solvating disordered DMSO molecules included in the crystal network are located between the chains and will fulfill the function of a hydrophobic surface “smoother” (Figure 9d). The consequence of such a crystal structure of complex **3** is its low stability.

### 3.6. FT-IR and FT-Raman Spectroscopy

**Figure 10:**
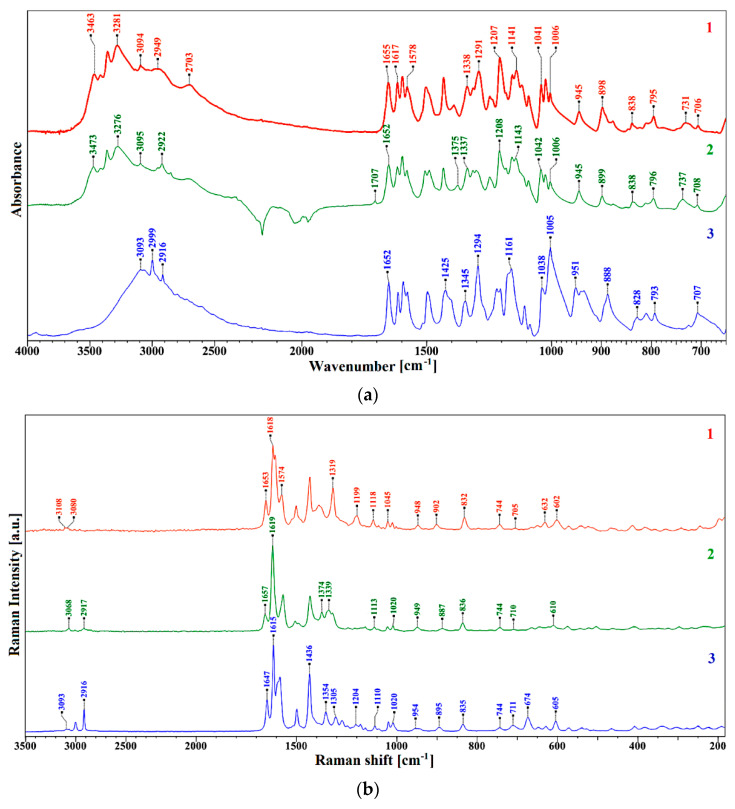
(**a**) FTIR-ATR and (**b**) FT-Raman spectra of complexes **1**–**3**.

The FTIR-ATR and FT-Raman spectra in solid state for studied complexes **1**–**3** are presented in Figure 10. The IR spectra of these complexes show a very broad band within the spectral range of 3500–2500 cm^−1^ with a few non-resolved peaks on the band envelope (Figure 10a). These bands are attributed to the stretching vibrations ν(O–H) of structural water molecules in the cation coordination sphere and H-bonded phenolic groups. The bands corresponding to the bending mode of those groups appear between 1600 and 1300 cm^−1^. In this range, the stretching vibrations ν(=C–H) of rings and ν(C–H) solvated solvents can also be found. The band centred at 3095 cm^−1^ represents common stretching vibrations in aromatic rings while the weak and sharp signals at 2992, 2922, and 2916 cm^−1^ correspond to the ν(C–H) stretching mode of the solvent methyl group in the IR spectrum of **2** and **3**, respectively. The coordination of water ligand in the crystal lattice of **1** was confirmed by the prominent bands at 3463–3281 cm^−1^, 2929 and 2703 cm^−1^.

The IR spectra of solvates show the intensive bands at 3473–3276 and 2703 cm^−1^ which confirmed the presence of water molecules in crystal **2**, while the bands in 3 completely disappeared. Since the profile of these bands in **1** and **2** are very close to each other, it may indicate a similar arrangement of water molecules in the cation coordination sphere. It may also suggest that some amounts of acetone molecules intercalate into the crystal network of complex **2**, which may explain very low stability of these crystals. Moreover, the changes in the IR spectrum for complex **3** unambiguously show the complete exchange of water molecules for DMSO during the solvothermal reaction. In the Raman spectra of this range, only very weak stretching vibrations ν(=C–H) and ν(C–H) are observed at 3108–3080 cm^−1^, 3068–2917 cm^−1^, and 3093–2916 cm^−1^ for **1**–**3**, respectively (Figure 10b). This is due to the lack of signals from the polar phenolic OH groups and water molecules.

The occurrence of acetone molecules in complex **2** is confirmed by the presence of a very weak IR band of the carbonyl groups ν(C=O) at 1707 cm^−1^, i.e., very close to the natural vibration frequency of this group in pure acetone (~1714 cm^−1^). This further supports the hypothesis about the location of acetone as a solvating molecule. Information on the acetone inclusion into crystal **2** is also supplemented by the presence of the characteristic IR absorption bands relating to the symmetrical stretching and bending vibrations of the –CH_3_ groups at 2922 and 1375 cm^−1^, as well as the corresponding bands in the Raman spectrum (RS) at 2917 and 1374 cm^−1^.

The presence of DMSO in crystal **3** is evidenced by intense bands of asymmetric and symmetric stretching vibrations of methyl groups –CH_3_ at 2999, 2916 cm^−1^ (IR) and 3002, 2916 cm^−1^ (RS) as well as deformation bands at 1425, 1390 cm^−1^ (IR) and 1436, 1305 cm^−1^ (RS). However, the most convincing evidence for the presence of DMSO in crystal **3** is very intense IR bands of stretching vibrations ν(S=O) at 1038 and 1001 cm^−1^ related to the solvent molecules and these coordinated to potassium cation, respectively.

The most diagnostic region in the IR and Raman spectra at 1657–1300 cm^−1^ includes the stretching vibrations of carbonyl groups ν(C=O), the bending vibration of phenolic group δ(O–H) and the common bands of skeletal ring vibrations ν(C–C). The skeletal vibrations are coupled with the δ(C–H) and δ(OH) bending deformations of rings [55]. Moreover, the bands attributed to water molecules occur in this region of the IR spectrum. As a result, the IR bands of the complexes **1**–**3** are broadened and have a complex shape. In contrast, the Raman spectra in this region are characterized by sharp and separated peaks due to the lack of signals from polar water molecules. Generally, the QSA¯ anion contains five phenolic OH groups but their vibrations should be different as the C5–OH and C3–OH hydroxyl groups participate in intramolecular interactions with the C=O group as well as both hydroxyl groups C3’–OH and C4’–OH in the B ring. Moreover, the hydroxyl group in the C7–OH position can form intermolecular hydrogen bonds. The different nature of the hydroxyl groups is confirmed by the IR and Raman spectra [11,56,57].

In all crystal structures, the carbonyl group is a double acceptor of intramolecular hydrogen bonds in the O5-H…O4…H-O3 moiety, which also coordinate K^+^ cation (Figure 2), so the stretching vibration of C=O groups in the IR and Raman spectra of **1**–**3** are very similar: 1655 (**1**), 1652 (**2**), and 1652 cm^−1^ (**3**). In the Raman spectra, the corresponding bands are centered at slightly lower wavenumbers: 1653, 1657, and 1647 cm^−1^, respectively. The location of the stretching ν(C=O) vibrations well below 1700 cm^−1^ indicates strong interactions with the nearest surroundings (Figure 2). Thus, the obtained results suggest that, similarly to **1** and **3**, the carbonyl group in complex **2** is located in the coordination sphere of K^+^. The skeletal ring stretching modes give bands in the range of 1619–1560 cm^−1^ (IR, RS). Intense signals in the IR and Raman spectra related to the δ(O–H) bending modes of phenolic groups and combination of this with ν(C–O) stretching vibration are also observed in the region.. The corresponding strong and very strong bands of these modes are observed in the IR spectra at the following ranges: 1578–1338 cm^−1^ (**1**), 1579–1337 cm^−1^ (**2**), and 1578–1345 cm^−1^ (**3**). In the Raman spectra, these groups give bands at 1574–1319, 1568–1399, and 1583–1354 cm^−1^, respectively. The next spectral region between 1300 and 900 cm^−1^ corresponds to the ν(C–O) and ν(C–O–C) vibration modes. This area also includes the coupled ν(S=O) stretching and ν(S–O) vibrations of sulfonic groups. The strong bands of asymmetric and symmetric ν(S=O) vibrations were observed at the following wavenumbers: 1291, 1175, and 1141 (**1**); 1208, 1158 and 1144 cm^−1^ (**2**); 1294, 1161 cm^−1^ (**3**). The corresponding ν(S–O) vibrations were placed at 1042, 1024, and 1006 cm^−1^ (**1**); 1042, 1025, and 1006 cm^−1^ (**2**); 1038 and 1006 cm^−1^ (DMSO) (**3**). The ν(C–S) absorption bands in the IR and Raman spectra are usually placed at 800–600 cm^−1^ region. However, the ν(S=O) and ν(S–O) bands in the Raman spectra were completely invisible or of a very weak intensity due to the strong polarity of this group. The above comparison shows that the solid state spectra are consistent with the presented crystal structures, and the vibrations of all molecular fragments converge the specific profiles of the IR and Raman spectra. Spectral band assignments are also consistent with earlier descriptions for quercetin and its sulfonic derivatives [35,58,59,60,61,62,63,64].

### 3.7. Desolvation of Crystals ***1*** and ***3*** in a Vacuum

**Figure 11:**
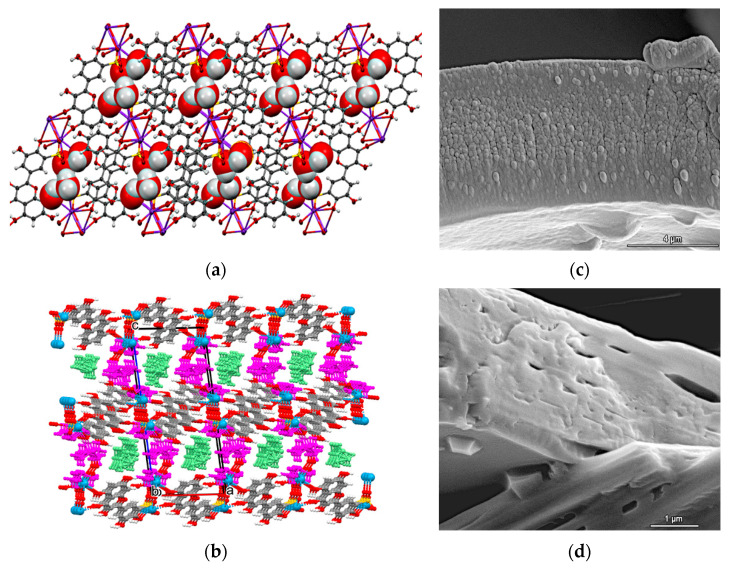
(**a**,**b**) Location of water and DMSO molecules in the net of crystals **1** and **3**, respectively. In **3**, solvating DMSO molecules are illustrated in green and coordinating ones—in magenta. (**c**,**d**) SEM micrographs under vacuum showing effects of solvent evaporation from the crystal network for **1** and **3**, respectively.

During the SEM measurements in high vacuum mode, a slow dehydration/desolvation process of the crystals was observed due to the presence of solvents in the network of **1** and **3** (Figure 11). This phenomenon for **1** is manifested by a huge amount of bubbles and micro-slits on the crystal surface (Figure 11c). The evaporation of the solvent from the layered crystal structure in **3** occurs in the form of numerous visible deformations and perforations along those planes (Figure 11d). Thus, the observed effects are consistent with the supramolecular structure of the analyzed crystals.

## 4. Conclusions

The crystal structure of potassium quercetin-5′-sulfonate complexes: **1** (diaqua) and **3** (DMSO) was successfully established, while **2** (acetone-solvated) crystals proved to be extremely unstable in air and their structure was not solved. Since complex **2** is very unstable, it was only possible to take images of the crystal morphology and the IR and Raman spectra. This only allows for hypotheses about the probable structure of the complex **2** in the solid phase.

The quercetin-5′-sulfonate anion is a multidentate *O*-ligand and is heptadentate one in complexes **1** and **3**. The formation of KO_8_ coordination polyhedrons involves sulfonic, phenolic, and carbonyl groups of anions and two neutral molecules (H_2_O or DMSO). It is interesting that the same set of anion O-atoms is involved in the coordination of the K^+^ cations of both complexes and K_2_O_14_ dimers of polyhedra are formed. However, different combinations of the chelating or bridging functions of these atoms result in the formation of a three-dimensional polymer in one case (1—[KQSA(H_2_O)_2_]) and a linear one-dimensional polymer in the other (**3**—[KQSA(DMSO)_2_]DMSO). This is due to the influence of the solvents used in the synthesis. The larger and more hydrophobic DMSO molecule weakens the anion–anion contacts and causes their separation, which requires filling the free spaces in the network by intercalating additional DMSO molecules.

The evaporation of the solvent from the space between the polymeric layers occurs in high vacuum, which causes deformations and perforations of the faces of crystals **1** and **3**. This effect additionally confirms the process of solvent intercalation into the crystal networks.

The IR and Raman spectra of the studied crystals **1** and **3** are consistent with their molecular structure. Since the IR spectra profiles of **1** and **2** are very similar to each other, it may indicate the presence of water molecules in **2** and an analogous arrangement in the cation coordination sphere. At the same time, it can be assumed that included acetone molecules are located in some voids of the crystal and are not coordinated to K^+^.

The thermal analysis revealed that only **1** is thermally stable up to about 50 °C, and its decomposition process in air and helium atmosphere is multistage. The first stage of its degradation, between 50–200 °C with maximum rates at 127 °C (helium) and 144 °C (air), is always associated with the loss of coordinated water molecules. The TG–DTG-DSC and EGA analyses showed that an organic moiety decomposition of **1** takes place in one (helium—at 350 °C) or two (air −351 and 449 °C) steps. The TG–EGA analysis indicated that the first oxidative step (300–400 °C) corresponds to the detachment of the sulfone group and the breakdown of the aromatic ring B of QSA¯ anion. In the second stage (400–600 °C) apart from the ring B disintegration, the breakdown of the aromatic rings A and C in the QSA¯ moiety also occurs. In helium, the disintegration and pyrolysis process of all rings (A, B and C) take place simultaneously. On the basis of TG–DTG–DSC runs and powder XRD patterns, it was proved that—in an oxidative atmosphere—thermal decomposition process is completed up to 600 °C and the final product of thermal degradations of the complex is K_2_SO_4_. On the other hand, the pyrolysis of **1** in helium is not completed at 1400 °C and a mixture of K_2_C_2_ and soot is a probable product of the thermal degradation at 800 °C.

Studies have shown that the solubility of quercetin in water can be increased after chemical modification of the molecule, for example by sulfonation and complexation with potassium ions.

## Figures and Tables

**Table 1:** The characteristic parameters of complexes of sulfonated flavonoids with metal cations. Refcodes for the crystal structure analyses were taken from the Cambridge Structural Database.

Cation	CSD Refcode/SO_3_^−^ Substitution	Coordination Sphere	Coordinating SO_3_^−^ Group	Type of Complex	Reference
Na	DURTEQ/C8	CN = 6, NaO_6_	yes	1D polymer	[17]
	SULFOV/C8	CN = 5, NaO_5_ CN = 5, NaO_5_	yes	3D polymer	[18]
K	QOLLAF/C8	CN = 5, KO_5_	yes	3D polymer	[19]
Ca *	POMDUR/C8	CN = 7, CaO_7_	yes	mononuclear	[20]
Ba	PEHROK/C6	CN = 10, BaO_10_	yes	1D polymer	[21]
Mn *	YELTAN/C6	CN = 6, MnO_6_	no	mononuclear	[22]
Co *	WIZGIW/C6	CN = 6, CoO_6_	no	mononuclear	[23]
Ni *	WEPPAJ/C6	CN = 6, NiO_6_	no	mononuclear	[24]
	YELSUG/C6	CN = 6, NiO_6_	no	mononuclear	[22]
Cu *	YIGZOE/C6	CN = 5, CuO_5_	no	mononuclear	[25]
Ag	PAGVUS/C6	CN = 4, AgO_4_ CN = 4, AgO_4_	yes	tetranuclear	[26]
Zn	ZUJTIJ/C5′	CN = 6, ZnO_6_	yes	1D polymer	[27]
Cd	XESPES/C6	CN = 7, CdO_7_ CN = 7, CdO_7_ CN = 7, CdO_7_ CN = 7, CdO_7_	yes	tetranuclear	[28]
Na, Zn	ZUJGUI/C5′	CN = 6, ZnO_6_ CN = 6, NaO_6_	no yes	1D polymer 1D polymer	[27]

CN—coordination number; *—aqua complex.

**Table 2:** The geometry of the cation K^+^ coordination sphere in **1**.

K-O	Symmetry Transformations	d(K-O) [Å]
* K(1)-O(4’)#2	#1 −x, −y + 1, −z + 1 #2 −x, −y, −z + 2 #3 x − 1, y − 1, z + 1	3.016(2)
*$ K(1)-O(4’)		3.101(2)
**$** K(1)-O(2S)		2.754(2)
K(1)-O(1S)#2		2.754(2)
K(1)-O(4)#1		2.713(2)
K(1)-O(5)#3		2.759(2)
K(1)-O(1W)		3.034(2)
K(1)-O(2W)		3.029(2)

*—common edge of polyhedra; **$**—chelating fragment.

**Table 3:** Geometry of O-H…O hydrogen bonds for crystal **1**.

D-H...A	d(D-H) [Å]	d(H...A) [Å]	d(H...A) [Å]	<(DHA) [°]
O(1W)-H(2W1)…O(2W)	0.89(5	2.05(5)	2.849(3)	150(3)
O(1W)-H(1W1)…O(2S)	0.83(4)	2.03(4)	2.766(2)	147(3)
O(4’)-H(4’)...O(3’)	0.77(3)	2.21(3)	2.653(2)	118(2)
O(3)-H(3)...O(4)	0.84(3)	2.29(3)	2.718(2)	112(2)
O(5)-H(5)...O(4)	0.88(3)	1.78(3)	2.610(2)	156(3)
O(2W)-H(1W2)...O(3S)#*a*	0.83(4)	2.02(4)	2.829(2)	164(3)
O(2W)-H(2W2)...O(3S)#*b*	0.92(4)	1.90(4)	2.814(2)	173(3)
O(3’)-H(3’)...O(1W)#*c*	0.86(3)	1.78(3)	2.628(2)	169(3)
O(4’)-H(4’)...O(7)#*d*	0.77(3)	2.09(3)	2.764(2)	146(3)
O(7)-H(7)...O(1S)#*e*	0.83(3)	1.98(3)	2.783(2)	161(3)
O(3)-H(3)...O(2W)#*f*	0.84(3)	2.03(3)	2.823(2)	158(2)

Symmetry transformations used to generate equivalent atoms: #*a* x − 1, y, z #*b* −x, −y, −z + 1 #*c* x, y + 1, z #*d* x − 1, y, z + 1 #*e* −x + 1, −y, −z + 1 #*f* −x, −y + 1, −z + 1.

**Table 4:** The thermogravimetric analysis data of **1** in helium and air (where: Δm—mass loss; t_peak_—peak temperature on the DSC curve; T_DTG(max)_—temperature at maximum mass loss on the DTG curve). Effects: (−) endo, (+) exo.

Medium	Stage	t_peak_ [°C]	Thermogravimetry		
			Temp.Range; T_DTG(mx)_ [°C]	Δm [%] Found (calc.)	Residue [%] Found (calc.)
air	I	154 (−)	25−300; 144	8.24 (7.89)	19.31 (19.09) 0.5 K_2_SO_4_
		280 (+)			
	II	367 (+)	300−400; 351	20.00	
	III	453 (+)	400−600; 449	52.45	
	IV	1062 (−)	800−1400; 1215	14.13	5.18
helium	I	130 (−)	25−300; 127	8.13 (7.89)	42.79 (42.72) 0.5 K_2_C_2_ + 12C
		280 (+)			
	II	350 (−)	300−700; 350	49.08	
	III		700−1420; -	29.30	13.49

**Table 5:** The solubility of QUE·2H_2_O and **1** at 20 °C.

Solvent	QUE·2H_2_O		1	
	[g/L]	[mol/L]	[g/L]	[mol/L]
Water	0.002	5.91·10^−6^	0.09	1.97 × 10^−4^
Acetone	3.69	1.10·10^−2^	0.25	5.48 × 10^−4^
Ethanol	8.11	2.40·10^−2^	0.06	1.31 × 10^−4^
DMSO	685	2.03	167	0.37

**Table 6:** The geometry of the cationic coordination spheres in **3**.

K(1)-O	Symmetry Transformations	d(K-O) [Å]	K(2)-O	Symmetry Transformations	d(K-O) [Å]
K(1)-O(1C)	#1 1 − x, 1 − y, −z #2 2 − x, 2 − y, −z #3 x + 1, y + 1, z	2.672(5)	K(2)-O(5B)	#4 1 − x, 2 – y, 1 – z #5 −x, 1 − y, 1 – z #6 x − 1, y − 1, z	2.685(5)
K(1)-O(1E)		3.037(7)	K(2)-O(3SB)#4		2.657(5)
K(1)-O(5A)		2.730(5)	K(2)-O(4’B)#4		2.934(5)
* K(1)-O(2SA)#3		2.807(6)	K(2)-O(4B)#5		2.725(5)
*$$ K(1)-O(2SA)#1		2.661(5)	K(2)-O(3SB)#6		2.796(6)
**$** K(1)-O(4’A)#1		2.835(5)	K(2)-O(1SB)#6		3.019(5)
**$** K(1)-O(1SA)#3		3.167(6)	K(2)-O(1F)		2.681(6)
K(1)-O(4A)#2		2.675(5)	K(2)-O(1D)		2.962(7)

*—common edge of polyhedra; **$**—chelating fragment.

**Table 7:** Geometry of O-H…O hydrogen bonds observed in crystal **3**.

D-H...A	d(D-H) [Å]	d(H...A) [Å]	d(H...A) [Å]	<(DHA) [°]
O(3A)-H(3A)...O(4A)	0.84	2.23	2.688(6)	115
O(5A)-H(5A)...O(4A)	1.19	1.65	2.671(7)	139
O(4′A)-H(4′A)...O(3′A)	0.90	2.14	2.695(7)	112
O(7A)-H(7A)...O(3SA)#*a*	0.79	1.90	2.673(7)	163
O(3A)-H(3A)...O(1C)#*b*	0.84	2.04	2.770(7)	144
O(4′A)-H(4′A)...O(1E)#*c*	0.90	1.98	2.653(7)	130
O(3′A)-H(3′A)...O(1G)	0.84	1.82	2.661(8)	177
O(3B)-H(3B)...O(4B)	0.96	2.06	2.687(7)	121
O(5B)-H(5B)...O(4B)	0.93	1.86	2.665(7)	144
O(7B)-H(7B)...O(2SB)#*d*	0.88	1.84	2.697(7)	165
O(4′B)-H(4′B)...O(1D)#*e*	0.88	1.90	2.649(8)	142
O(3B)-H(3B)...O(1F)#*f*	0.96	2.05	2.796(7)	133
O(3’B)-H(3’B)...O(1H)#*e*	0.84	1.85	2.684(9)	172

Symmetry transformations used to generate equivalent atoms: #*a* −x, −y + 1, −z #*b* −x + 2, −y + 2, −z #*c* −x + 1, −y + 1, −z #*d* −x + 2, −y + 2, −z + 1 #*e* −x + 1, −y + 2, −z + 1 #*f* −x, −y + 1, −z + 1.

## Data Availability

The data presented in this study are available on request from the corresponding author.
